# *Giardia duodenalis* in faecal samples from calves in Poland

**DOI:** 10.2478/jvetres-2025-0052

**Published:** 2025-09-24

**Authors:** Jacek Sroka, Jacek Zwoliński, Ewa Bilska-Zając, Angelina Wójcik Fatla, Jacek Karamon, Jolanta Małgorzata Zdybel, Weronika Wiktoria Korpysa-Dzirba, Małgorzata Samorek-Pieróg, Weronika Piotrowska, Tomasz Cencek

**Affiliations:** 1 Department of Parasitology and Invasive Diseases, Bee Diseases and Aquatic Animal Diseases, National Veterinary Research Institute, 24-100 Puławy, Poland; 2 Department of Health Biohazards and Parasitology, Institute of Rural Health, 20-090 Lublin, Poland

**Keywords:** cattle, *Giardia duodenalis*, molecular characterisation, PCR

## Abstract

**Introduction:**

*Giardia duodenalis* is the prevalent parasitic protozoan responsible for diarrhoeal disease in humans and animals. This study aimed to determine the prevalence and assemblages of this parasite present in cattle in Poland.

**Material and Methods:**

Faecal samples were collected from 1,602 cattle up to 4 months old, bred on 267 farms located in all 16 voivodeships of Poland. Extraction of DNA was performed using a modified alkaline and a heat lysis method. Molecular detection of *β-giardin* gene by PCR and sequence analysis were performed.

**Results:**

In 174 of 1,602 (10.9%) tested cattle and in 89 of 267 examined herds (33.3%), *G. duodenalis* DNA was detected. The highest prevalence of *Giardia* in cattle was found in the Lubelskie (25%), Mazowieckie (21.3%) and Zachodniopomorskie (20.4%) voivodeships. In other regions, the prevalence did not exceed 10%. The number of *Giardia*-positive cattle decreased with animal age. The most frequently identified assemblage in cattle was E (89.2%), and A and B were detected more seldom in 9.0% and 1.8% of cattle, respectively. No significant differences in positive results were observed in cattle depending on production purpose.

**Conclusion:**

The study results showed a high prevalence of *Giardia* in cattle in Poland. The presence of the *Giardia* A and B assemblages indicates a potential zoonotic threat.

## Introduction

*Giardia duodenalis* (syn. *G. intestinalis* and *G. lamblia*) is one of the most common protozoan parasites causing diarrhoeal disease in humans and companion animals, livestock and wildlife worldwide (8, 11, 21). The prevalence of *Giardia* infection in people was estimated at 2–5% in industrialised countries and 20–30% in developing countries (15). The risk of disease is increased in children, immunocompromised people and the elderly. In Poland between 2022 and 2024, 1,340–2,150 cases of giardiasis in humans were reported annually, which corresponds to 3.5–5.7 cases per 100,000 inhabitants (31, 32).

*Giardia* prevalence in cattle varies with farm management systems, animal husbandry practices and geographic and climatic factors. Infection with this parasite may to be equally prevalent in dairy and beef cattle. Risk factors considered significantly associated with cattle *Giardia* infection were the type of flooring (26, 27) and housing, management practices and direct contact with infected animals (12). The transmission of *Giardia* parasites can be between infected and healthy calves in the acute or chronic phase of infection; asymptomatic adults may also be a source of disinfectantresistant *Giardia* cysts (6, 14, 28, 34). In calves, *Giardia* can induce diarrhoea, impair absorption of fluids, nutrients and electrolytes and cause dehydration, poor condition and a reduction in weight (3). Negative impacts may also be felt off the farm: infected calves can figure significantly as threats to public health in the forms of sources of water contamination (9). These parasites are transmitted to humans by the faecal–oral route through contaminated water and food or by direct contact between animals and humans as well as (less commonly) between humans (14).

The risk to humans of *G. duodenalis* infection developing disease is not universal to all of its at least eight genetically distinct assemblages (currently known to be A–H). Only assemblages A and B are considered potentially zoonotic (24, 37). In cattle, assemblage E is the most prevalent, followed by assemblage A (12). However mono- and mixed infections with assemblage A or assemblage B were also reported in cattle (14). Infection with assemblages C, D and F were also detected in cattle, sheep and pigs in the United Kingdom (30) and Spain (7). In contrast to the good availability of data on the prevalence of giardiasis in cattle in some European countries (12), only limited data is available about the epidemiology of *G. duodenalis* in cattle in Poland. The objective of the present study was to undertake molecular characterisation of *Giardia duodenalis* in calves in Poland with regard to the circulating assemblages and to investigate the parasite’s prevalence in these livestock.

## Material and Methods

Faecal samples of 10–15 g mass were collected from 1,602 cattle located in all 16 voivodeships of Poland. Animals were divided into four age groups: 1–3 weeks, 4–6 weeks, 7–9 weeks and 10–16 weeks. Upon collection, faeces were placed individually into plastic containers, labelled and sent to the laboratory of the Department of Parasitology and Invasive Diseases, Bee Diseases and Aquatic Animal Diseases, at the National Veterinary Research Institute in Puławy. Farms were randomly selected in each region. Age, breed and animal sex data required to estimate the potential risk factors for *G. duodenalis* infection in cattle herds was collected using a questionnaire. According to notes provided by herd veterinarians, the animals did not show any symptoms of diseases and were in good health.

The animals were housed on 267 farms, and managed in herds averaging 137 head. There were 175 large farms (65.5%) with >50 cattle and 92 small farms (34.5%) with <50 cattle. The tested animals represented 14 breeds, mainly dairy (74.8%), and in far smaller proportions beef (14.5%) and mixed (10.7%) breeds. The majority of the calves were Holstein (n = 944), nearly one tenth were mixed beef breeds (n = 142), and the remainder were Polish Black and White (n = 99), Simmental (n = 87) and Polish Red and White Holstein– Friesian (n = 69). The numbers of cattle from individual voivodeships and the *Giardia* prevalence analysed in three age groups are presented in Table 1.

**Table 1.** Voivodeship distribution of *Giardia duodenalis* DNA in cattle in Poland

Voivodeship	Animals tested (n)	PCR^+^ animals (n)	Prevalence (%)	*Giardia duodenalis* assemblage (n (%))		
				A	B	E
LS	108	9	8.3	1 (11.1)	-	8 (88.9)
PM	108	7	6.5	1 (14.3)	-	6 (85.7)
ZP	108	22	20.4	1 (4.8)	-	20(95.2)
OP	72	4	5.6	1 (33.3)	-	2 (66.7)
DŚ	90	0	0	-	-	-
ŚL	72	7	9.7	1 (25.0)	-	3 (75.0)
PK	72	3	4.2	1 (33.3)	-	2 (66.7)
ŚK	108	7	6.5	-	-	7 (100)
ŁD	108	7	6.5	-	1 (14.3)	6 (85.7)
MP	108	15	13.9	-	1 (6.7)	14 (93.3)
LB	108	27	25.0	5 (20.0)	-	20 (80.0)
MZ	108	23	21.3	1 (4.3)	-	22 (95.7)
PD	108	14	13.0	2 (15.4)	1 (7.7)	10 (76.9)
WP	108	14	13.0	-	-	14 (100)
WM	108	6	5.6	1 (16.7)	-	5 (83.3)
KP	108	9	8.3	-	-	9 (100)
Total	1,602	174	10.9	15 (9.0)	3 (1.8)	148 (89.2)

1 LS – Lubuskie; PM – Pomorskie; ZP – Zachodniopomorskie; OP – Opolskie; DŚ – Dolnośląskie; ŚL –Śląskie; PK – Podkarpackie; ŚK – Świętokrzyskie; ŁD – Łódzkie; MP – Małopolskie; LB – Lubelskie; MZ – Mazowieckie; PD – Podlaskie; WP – Wielkopolskie; WM – Warmińsko-Mazurskie; KP – Kujawsko-Pomorskie; χ^2^ = 74.02; P-value < 0.001

Extraction of DNA from stool samples (0.1 g) was performed using a modified alkaline and a heat lysis method according to Millar *et al*. (29), combined with an additional step of 15 cycles of freezing in liquid nitrogen and thawing to maximise cyst lysis (33). DNA were purified using a GeneMATRIX PCR/DNA Clean-Up Purification Kit (EURx, Gdańsk, Poland) according to the manufacturer’s instructions and the DNA was stored at –20°C until use.

A semi-nested PCR according to Caccio *et al*. (4) with slight modifications was performed for molecular identification of *Giardia* spp. by the *β-giardin* gene. The reaction mixture had a volume of 50 μL and comprised 3.2 mM of MgCl_2_, 0.1 μM of each primer, 1.3 units of Taq polymerase (Qiagen, Hilden, Germany), 0.2 mM of each deoxynucleoside triphosphate (Fermentas, Vilnius, Lithuania) and 2.5 μL of DNA. The PCR conditions were as follows: an initial denaturation step at 94°C for 5 min; followed by 40 cycles of 94°C for 30 s, 65°C for 30 s and 72°C for 60 s; and a final extension at 72°C for 7 min. Each semi-nested PCR product was subjected to electrophoresis on 1.5% agarose gel and stained with ethidium bromide.

The obtained sequences were compared with reference sequences using the National Center for Biotechnology Information Basic Local Alignment Search Tool. The GenBank database reference sequences in the comparisons were KF963547.1 (sub-genotype A1), AY072723 (A2), AY072724 (A3), AY072725 (sub-genotype B1), AY072726 (B2), AY072727 (B3), AY072728 (B4), JF422718 (assemblage C), HQ538709 (assemblage D), AY072729 (assemblage E) and AY647264 (assemblage F).

### Statistical analyses

A chi-squared (χ^2^) test with Yates’ correction was used to analyse differences in regional, age- and breed-correlated *Giardia* prevalence in cattle. Probability values of <0.05 were considered significant.

## Results

**Fig. 1.**
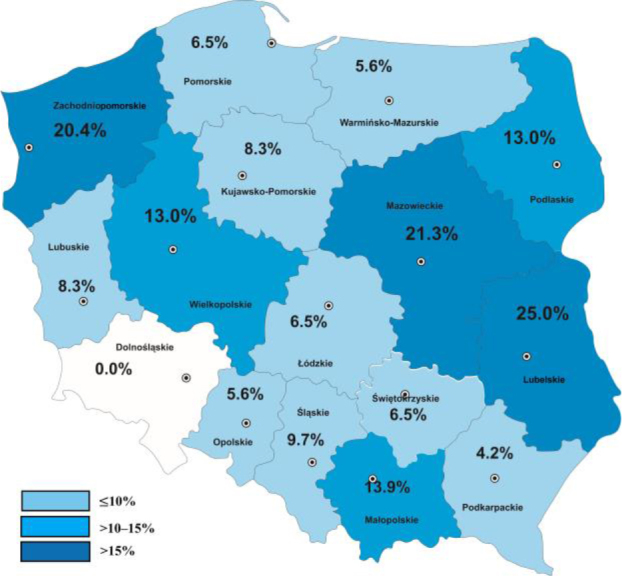
Prevalence of *Giardia duodenalis* DNA in cattle by Polish voivodeship

DNA of *G. duodenalis* was detected in 174 of the 1,602 (10.9%) tested cattle and in 89 of the 267 examined herds (33.3%). The highest prevalence of *Giardia* in cattle was found in the Lubelskie (25.0%), Mazowieckie (21.3%) and Zachodniopomorskie (20.4%) voivodeships, less prevalence was found in the Małopolskie (13.9%) and Wielkopolskie (13.0%) voivodeships. In the others the prevalence did not exceed 10%. The lowest percentages were noted in the Podkarpackie (4.2%) and Warmińsko-Mazurskie (5.6%) (P-value <0.001). In the Dolnośląskie voivodeship, no positive results were found (Fig. 1, Table 1). The inequality of distribution of positive results among voivodeships was statistically significant (χ^2^ = 74.02; P-value < 0.001).

**Fig. 2.**
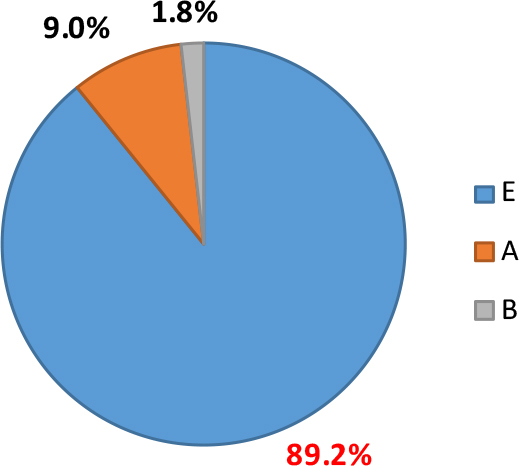
Distribution of *Giardia duodenalis* assemblages among positive isolates from Polish cattle

The prevalence of *Giardia* assemblages was identified by DNA sequence analysis of the *β-giardin* gene for selected PCR-positive samples. The most frequent *G. duodenalis* assemblage identified in cattle was E (89.2%). The assemblages with zoonotic potential – A and B – were detected in 9.0% and 1.8% of cattle, respectively (Fig. 2).

All breeds except mixed dairy, Charolaise and Red Polish were positive for *Giardia*. The highest percentages of positive results were found for Belgian Blue × Brown Swiss × Jersey crossed breeds (16.7%), Limousin (16.2%) and mixed beef (15.5%). The lowest prevalence among the positive breeds was found for Polish Black and White Holstein-Friesian crossed with diverse breeds (2.5%) and Montbéliarde (9.1%). Taking into account the breeds of which there were highest number of animals, statistically significant differences (P-value < 0.05) were found between mixed beef (15.5%) and Polish Black and White Holstein-Friesian (9.9%) and between mixed beef and Polish Black and White Holstein-Friesian crossed with diverse breeds (2.5%) (Table 2).

**Table 2.** Occurrence of *Giardia duodenalis* in individual breeds of Polish cattle

Cattle breed	Animals tested (n)	PCR^+^		Assemblage (n (%))			Unidentified (n)
		Number	%	A	B	E	
HO	944	93	9.9	10 (11.0)	1 (1.1)	80 (87.9)	2
MB	142	22	15.5	-	-	17 (100)	5
ZB	99	13	13.1	1 (7.7)	1 (7.7)	11 (84.6)	-
SM	87	9	10.3	2 (22.2)	-	7 (77.8)	-
RW	69	7	10.1	-	-	6 (100)	1
LM	37	6	16.2	1 (16.7)	1 (16.7)	4 (66.7)	-
MO	11	1	9.1	-	-	1 (100)	-
MS	8	0	0.0	-	-	-	-
CH	8	0	0.0	-	-	-	-
RP	7	0	0.0	-	-	-	-
HF×	40	1	2.5	-	-	1 (100)	-
BB, BS, JE	6	1	16.7	-	-	1 (100)	-
Others (No data)	143	21	14.7	1 (4.7)		20 (95.3)	-
Total	1,601	174	10.9	15 (9.0)	3 (1.8)	148 (89.2)	8

1 HO – Polish Black and White Holstein-Friesian; MB – Mixed beef breeds; ZB – Polish Black and White; SM – Simmental; RW – Polish Red and White Holstein-Friesian; LM – Limousin; MO – Montbéliarde; MS – mixed dairy breeds; CH – Charolaise; RP – Red Polish; HF× – HO mixed with one of SM, BD, LM, ZB, BB, MB, MO or SM; BB – Belgian Blue; BS – Brown Swiss; JE – Jersey

**Fig. 3.**
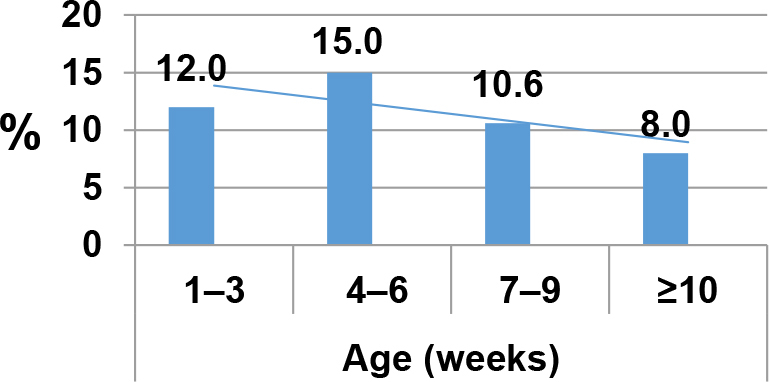
Association of *Giardia duodenalis* prevalence with age of cattle

The highest percentage of positive results for *Giardia* was found in cattle aged 4–6 weeks (15.0%) and a lower percentage in the age group ≥10 weeks (8.0%), and this difference was statistically significant (χ^2^ = 8.03, P-value < 0.01). Cattle aged less than 2 months were the majority host for *Giardia* and the number of positive cattle decreased with animal age (Fig. 3, Table 3). Age data were not available for 34 cattle; therefore the prevalence breakdown by age is of 1,568 animals instead of the 1,602 tested in total. Also, the age of one PCR-positive animal was unknown; therefore 173 results instead of 174 could be disaggregated by age.

**Table 3.** Association of *Giardia duodenalis* prevalence in young Polish calves with age

Age (weeks)	Animals tested (n)	Number of PCR^+^ animals (%)
1–3	251	30 (12.0)
4–6	412	62 (15.0)
7–9	330	35 (10.6)
≥10	575	46 (8.0)
Total	1,568	173 (11.0)

Among animals with available breed data, the majority were dairy breeds (67.6%), followed by mixed dairy/beef (14.5%) and beef breeds (17.9%). No significant differences in positive results were observed between cattle of different production purposes (Table 4).

**Table 4.** Association of *Giardia* prevalence with animal production system

Production purpose	Animals tested (n)	PCR^+^ animals (n (%))	Assemblage (n)			Unidentified
			A	B	E	
Dairy	1,063	111 (10.4)	13	2	92	4
dairy/beef	228	27 (11.8)	-	1	24	2
Beef	281	32 (11.3)	2	-	28	2
Total	1,572	170 (10.8)	15	3	144	8

## Discussion

The prevalence of *Giardia* in cattle varies depending on the geographical location, age of animals and the type of animal production, and the detected prevalence is different in different diagnostic methods (11, 30). Hygiene regimes, the type of water supply and farm management practices, including the stocking density of similar-age animals and the effectiveness of disinfection may also raise or reduce the exposure of cattle to *Giardia* (similarly to exposure to *Cryptosporidium*) (19). The prolonged intergravid periods found in dairy breeds may have a worsening effect on the infection rate. However, in this study the production type of cattle had no effect on the rate of *Giardia* infection.

Worldwide, the prevalence of *Giardia* in cattle ranged from 6.6% in New Zealand (23) to 58% in Canada and Australia (35). In Europe, the mean prevalence of *Giardia* in cattle was estimated at 35.1%; higher prevalence was recorded in neonatal calves (39.6%) than in adults (14.2%) (27). In Scottish beef and dairy cattle, the overall prevalence was 32.5%, being significantly higher in dairy (44.7%) than in beef cattle (10.1%) (1).

In the present work, *Giardia* DNA was successfully amplified from 10.9% (174 out of 1,602) of cattle faecal samples, which corresponds with the conclusions of other authors that *G. duodenalis* infections are common in calves in Europe (28). In our study, the highest prevalence of *Giardia* was found in cattle aged 4–6 weeks (15.0%) and the lowest in animals aged over 10 weeks (8.0%; P-value < 0.05). Bartley *et al*. (1) in Scotland found that 52.0% of five-week old calves were infected. Similar results were obtained by other authors: Hamnes *et al*. (16) in Norway found 49.0% of calves overall to be positive and 57.8% to be so in a group which were 2–3 months old. Guerden *et al*. (12) in multicentre study found the prevalence highest in calves of 5–8 weeks old and decreasing as animal age rose. Neonatal calves were almost free of *Giardia*. Huetink *et al*. (18) found only one infected calf in a group of 112 less than one month old in the Netherlands, and Oh *et al*. (36) detected *Giardia* cysts in 1.5% of calves younger than two weeks old in South Korea.

Several methods are used for *Giardia* detection in stool. Microscopy with trichrome staining has insufficient sensitivity in cases of low parasite density or when cysts are only excreted intermittently. Direct fluorescent assays using monoclonal antibodies specific to *Giardia* cysts have high sensitivity and specificity and are recommended as the gold standard for diagnosing giardiasis. Other methods include rapid immunochromatographic cartridge assays, enzyme immunoassays and the PCR-based tests of the kind used in the present study (17).

Among the genes that were used in previous research to confirm *Giardia* infection were mainly small subunit RNA, *β-giardin* and *GDH* (glutamate dehydrogenase). Sequencing of gene fragments was helpful in identifying assemblages (A to H). In cattle, *Giardia* DNA analysis most frequently identified assemblages E (also found in sheep and goats), A, which could be detected as its sub-genotypes AI–III, and B, characteristic for humans (14, 30). Likewise in this study, E, A and B *Giardia* assemblages were detected based on *β-giardin* gene sequence analysis. A significantly higher prevalence of *G. duodenalis* assemblage E (89.2%) than of assemblages A (9.0%) and B (1.8%) was found. The predominance of the E assemblage in cattle (based on *β-giardin* gene sequencing) was also reported in Italy (72%), the UK (71%) and Germany (56%); however, in France assemblage E was identified in a lower percentage of samples (39%) and assemblage A was predominant (61%) (12). The A and B assemblages could pose a zoonotic threat to humans, and their detection in calves might indicate an interaction between the human and livestock transmission cycles (40, 42). Assemblage B has been identified in cattle in China and Canada, and in dairy cattle it presented a larger potential zoonotic risk to humans of *Giardia* infection than it did in beef cattle (10, 25, 41). Among assemblage A *Giardia* detected in cattle in Europe, assemblage AII was revealed by sub-genotyping on the infrequent occasions when it was performed (37). Most studies did not confirm the zoonotic sub-type of assemblage A when detected in cattle (5). Bartley *et al*. (1) used three genes (*β-giardin, tpi* (triose-phosphate isomerase) and *GDH*) to analyse sequence data for assignment of assemblages and found that *β-giardin* was a good indicator of *Giardia* DNA, but for better analysis of sequences more than one locus was needed. Gillhuber *et al*. (13) used 18S ribosomal RNA (rRNA), *β-giardin* and *GDH* and found the 18S rRNA gene to be a good indicator of *Giardia* DNA, because the other genes were amplified only in about 10% and 5% of microscopically diagnosed cases.

The A (as sub-genotype AII) and B assemblages found in calves may be transmitted zoonotically (3), but other assemblages (C–F) may also occur in human cases of giardiasis (5). Langkjaer *et al*. (22) found zoonotic assemblage A in both young and older calves, and cows were infected mostly by isolates of assemblage E. The distribution of assemblages found in cattle differed from country to country, and the results of distribution investigations were dependent on the gene used in the research to detect them. In one genotyping study, using the *β-giardin* gene gave remarkably different results to those when the *tpi* gene was used (12). Bullumulla *et al*. (3) suggested six new genes that should be used in assemblage genotyping because of the unspecificity of the classical ones.

To our knowledge this is the first countrywide epidemiological study on *Giardia* prevalence conducted in Poland. Although research on *Giardia* in cattle has already been performed in Poland, it examined only small groups of animals (2, 39). It should be noted that the giardiasis in Poland is diagnosed mostly in children. It is likely that many cases are still undiagnosed, both in children and adults. Human giardiasis has been declining in Poland in the last decade, but when the number of registered cases declined to 358 in 2020, principally the impact of COVID-19 was seen (20). According to the National Institute of Public Health NIH - National Research Institute, 2,150 cases were registered in 2024, reaching the level noted before the pandemic (32). With human giardiasis cases returning to pre-pandemic levels in Poland, we consider understanding potential zoonotic reservoirs to have become more important and for utilisation of this understanding in developing comprehensive surveillance strategies to be prudent. The present research provides crucial baseline data on *Giardia* prevalence in cattle and documents the prevalence of potentially zoonotic assemblages A and B, pointing to the need for integrated human and animal surveillance systems.

## Conclusion

The results of the present study indicate high prevalence of *Giardia* on cattle farms in Poland. Young calves are especially at risk of infection, and several farm management factors are associated with this risk. The prevalence of assemblages A and B suggests a potential zoonotic threat, although further research is needed to understand better its potential impact on public health. It is also worth emphasising that in the Polish regions with a high rate of *Giardia* infection in cattle, a high number of recorded cases of giardiasis in humans was also noted (31), which may suggest that calves constitute a significant source of infection for humans in these regions.
